# A New Tool for Exploring Climate Change Induced Range Shifts of Conifer Species in China

**DOI:** 10.1371/journal.pone.0098643

**Published:** 2014-09-30

**Authors:** Xiaojun Kou, Qin Li, Carl Beierkuhnlein, Yiheng Zhao, Shirong Liu

**Affiliations:** 1 State Key Laboratory of Earth Surface Processes and Resource Ecology, Beijing Normal University, Beijing, PR China; 2 College of Life Sciences, Beijing Normal University, Beijing, PR China; 3 Department of Botany, University of British Columbia, Vancouver, B.C., Canada; 4 Department of Biogeography, University of Bayreuth, Bayreuth, Germany; 5 Institute of Forest Environment and Ecology, Chinese Academy of Forestry, Beijing, PR China; Institute of Botany, Chinese Academy of Sciences, China

## Abstract

It is inevitable that tree species will undergo considerable range shifts in response to anthropogenic induced climate change, even in the near future. Species Distribution Models (SDMs) are valuable tools in exploring general temporal trends and spatial patterns of potential range shifts. Understanding projections to future climate for tree species will facilitate policy making in forestry. Comparative studies for a large number of tree species require the availability of suitable and standardized indices. A crucial limitation when deriving such indices is the threshold problem in defining ranges, which has made interspecies comparison problematic until now. Here we propose a set of threshold-free indices, which measure range explosion (I), overlapping (O), and range center movement in three dimensions (Dx, Dy, Dz), based on fuzzy set theory (Fuzzy Set based Potential Range Shift Index, F-PRS Index). A graphical tool (PRS_Chart) was developed to visualize these indices. This technique was then applied to 46 Pinaceae species that are widely distributed and partly common in China. The spatial patterns of the modeling results were then statistically tested for significance. Results showed that range overlap was generally low; no trends in range size changes and longitudinal movements could be found, but northward and poleward movement trends were highly significant. Although range shifts seemed to exhibit huge interspecies variation, they were very consistent for certain climate change scenarios. Comparing the IPCC scenarios, we found that scenario A1B would lead to a larger extent of range shifts (less overlapping and more latitudinal movement) than the A2 and the B1 scenarios. It is expected that the newly developed standardized indices and the respective graphical tool will facilitate studies on PRS's for other tree species groups that are important in forestry as well, and thus support climate adaptive forest management.

## Introduction

The need to predict potential changes in species distribution in response to climate change has stimulated the development of Species Distribution Models (SDMs), or more generally named Ecological Niche Models (ENMs), which combine current species occurrence data with recorded environmental data layers to detect the distribution of and fundamental niche for a given species [1], [2], [3]. SDMs are reported to have moderate to high accuracy in reproducing current distributions based on training data sets [4]. Although still controversial regarding their transferability to future climates [5], [6], [7], [8], SDMs are the most effective and widely used approach to forecast future ranges of species [2].

In addition to the current progress in theoretical or methodological studies [9], [10], [11], [12], [13], SDMs have been increasingly applied in practice-oriented evaluations and assessments for climate adaptive management. This includes the conservation of endangered species [14], [15], [16], natural reserve networks [3], [10], [17], biodiversity changes [18], [19], [20], and climate change driven invasion processes [21]. In ecological or biodiversity studies, there is the trend that a large number of species are modeled to make inferences or test hypotheses. These species are chosen either from eminent taxonomical groups, or from major life forms of some regional species pools [20], [22], [23].

A systemic exploration of potential range shifts in response to future climates for large numbers of tree species is still not feasible (but see [4], [24], [25]). Studies on individual tree species range shifts are invaluable for climate adaptive forest management, but systematic studies on large groups of species or at least on a set of key species in a focal area are even more important. Most species are expected to experience range shifts to some extent; forest policy should understand the likelihood of changes by considering the sensitivity of species to a modified climate, the intensity of response, and the future availability of niche space. Identifying potential range changes, or ideally the patterns (spatially or taxonomically) of changes within a region, will guide forest policy in management [25].

Unlike in biodiversity studies, which are based on established indices for diversity and species turnover [23], species range studies lack standardized measures and quantitative indices. Approaches that are comparative, cumulative, or aggregated across species are less developed, however this is exactly what is needed in practice when a large set of species is concerned (as in forestry) and arbitrary preferences must be avoided.

SDM's can be realized for hundreds or thousands of species, but it is difficult to generalize results that subsume a large number of species projections. In addition, the projections are usually not illustrated in traditional binary map format but rather in continuous value format, which is suitable for representing potential habitat suitability or probability of occurrence [1]. Thresholds are adopted to convert these range maps to binary ones [23], [26], [27], [28].

The currently used threshold method makes interspecies comparisons of range shifts problematic. Firstly, how to best choose a threshold is still an unsolved problem. Furthermore, even if some standard species-based threshold selection procedure were widely accepted, the resultant thresholds would still be species specific, making comparisons among different species unreasonable. The development of taxonomical group level threshold selection procedure could be more complicated and is not foreseeable. Thus, a set of standard indices, which are threshold-free, are essential for both the researchers' and policy makers' perspectives. Fuzzy set theory is providing a promising theoretical concept for this purpose [29].

The adaptation of management directives to novel or changing conditions depends on successful communication between researchers and policy makers. Graphical representation is one of the most effective ways of facilitating interdisciplinary communication [30]. In the case of range shift studies, maps are a common method of graphical representation. However, preparing hundreds to thousands of individual maps for policy makers is not practical. Critical characteristics of range shifts and generality cannot be identified and the construction of simplified figures is challenging as well. It is difficult to summarize key patterns of range change and still maintain the information on individual species. Combining range shift indices with intuitive and easy-to-understand diagrams, based on precise data, would support the implementation of knowledge in climate adaptive forest management.

Due to the longevity of trees, forest management and adaptation must be proactively implemented as soon as possible if climatic features are changing. Conifers constitute perhaps the most important taxonomic group of trees in the world as much for their spatial extent as for their role in economy. This is also true for China, where ambitious afforestation programs are aiming at the maintenance and improvement of ecosystem services such as erosion control, water management, and the generation of biotic resources.

Pinaceae are representing the largest extant conifer family, including more than 230 species and with an extensive distribution [31], [32], [33], [34]. Most members of this family are trees that are usually dominant or co-dominant in their ecosystems [35]. The world's largest biome and ecosystem type, the boreal forests, is characterized by Pinaceae trees. Over a large scale, these conifers form the northern tree line and are thus of major importance when discussing temperature-dependent biome shifts. Pinaceae species also dominate mountain forests at lower latitudes in the Northern hemisphere, and are major components of temperate mixed forests [31], [33], [35]. Needle-leaved trees represent an important Plant Functional Type in global vegetation models [36], [37]. As mostly evergreen species (with the exception of the genus *Larix*), conifers are closely linked to climatic conditions throughout the year. Changing climate can affect conifers throughout all seasons.

Forests that are dominated by conifers are of global relevance in terms of economic resources, habitats for many species, and for the global carbon pool [31], [33], [35]. Thus, Pinaceae constitute one of the most important taxonomical groups in global forest ecosystems and in world forestry. Case studies on the future potential distribution of these trees are urgently needed.

In this study, we first project future ranges of the most abundant Chinese Pinaceae species using SDM's. We then develop and apply a quantitative index and a novel graphical tool to illustrate species potential range shifts. In a next step, we explore generality in the response of the whole group of Pinaceae species to changing climatic features. Finally, we discuss potential implications of this new approach in climate adaptive forest management.

## Materials and Methods

### Ethics statement

We confirm that no specific permissions were required for these study sites, the location is not privately-owned or protected in any way, and the field studies did not involve endangered or protected species.

### Diversity of the Pinaceae family in China

Eastern Asia is one of the most important origins for Pinaceae, and one of the major refuges during the Pleistocene glaciation periods for this family that is concentrated to the Holarctic realm. However, during their evolution, the Pinaceae family has experienced considerabe shifts in latitudinal and altitudinal distribution [38]. Today, China is a hotspot of species diversity for Pinaceae. Among the 11 genera of Pinaceae, the following genera are represented in China: *Abies*, *Keteleeria*, *Nothotsuga*, *Tsuga*, *Pseudolarix*, *Larix*, *Cathaya*, *Pseudotsuga*, *Picea*, and *Pinus*. Only the genus *Cedrus* is not represented. A great variety of Pinaceae species are distributed throughout the Chinese mainland, Taiwan and the Hainan islands, spanning tropical to subarctic climate zones [33], [35]. More than half (about 130 of 230) of the world's Pinaceae species occur in China [35].

Forests between the Great Xing'an Mountains and the Himalayas, and between Taiwan and the Altaic mountains, are dominated or co-dominated by Pinaceae species. In particular, four genera (*Abies*, *Picea*, *Larix* and *Pinus*) are common and species-rich in China, and constitute the major coniferous forests. The species of these four genera exhibit broad ranges in both geographical and environmental space. Single species are limited in their distribution areas, however, the taxonomic group is represented in across a wide range of climatic conditions. A clear geographical substitution sequence exists along the temperature gradient. For example, in the genus *Pinus*, *P. latteri*, *P. massoniana*, *P. tabulaeformis*, *P. koraiesis*, and *P. sylvestris* var. *mongolica* range from tropical to subarctic zones and exhibit a geographical substitution pattern [35]. The genus *Larix* is adapted to extremely cold temperatures during winter, as the species of this genus are deciduous and summer-green, avoiding frost damage especially in continental regions. Precipitation gradients are especially important at the margins of distribution, both as limiting factors of tree growth and as drivers of competition with other tree species.

### Species and environmental data

#### Species distribution data

We extracted distribution maps of the species from the digital version of the *Atlas of Chinese Vegetation 1∶1,000,000* [39] using ArcInfo Workstation version 8.3 (ESRI, Redlands, CA, USA). Within the four genera under focus, species were chosen that dominated or co-dominated forests based on a minimum threshold of thirty locations of presence. Rare and less abundant species were excluded in this study that is aiming towards ecosystem functions and forest management. A location of presence was defined as a 5′×5′ (geographical coordinates) cell that contained at least 1% forest area dominated or co-dominated by the target species. This treatment ensures that sample points are at least 5′ apart, without missing small patches (above 1% of the cell area) of species distribution within the cell. We modeled the distribution ranges where the species were dominant and disregarded the non-dominant distribution or scattered distribution areas of individual species. Whether a species dominated or co-dominated a forest was based on whether the vegetation type was named after the target species at the unit of ‘formation’, as defined in the *Vegetation of China* [35].

**Figure 1:**
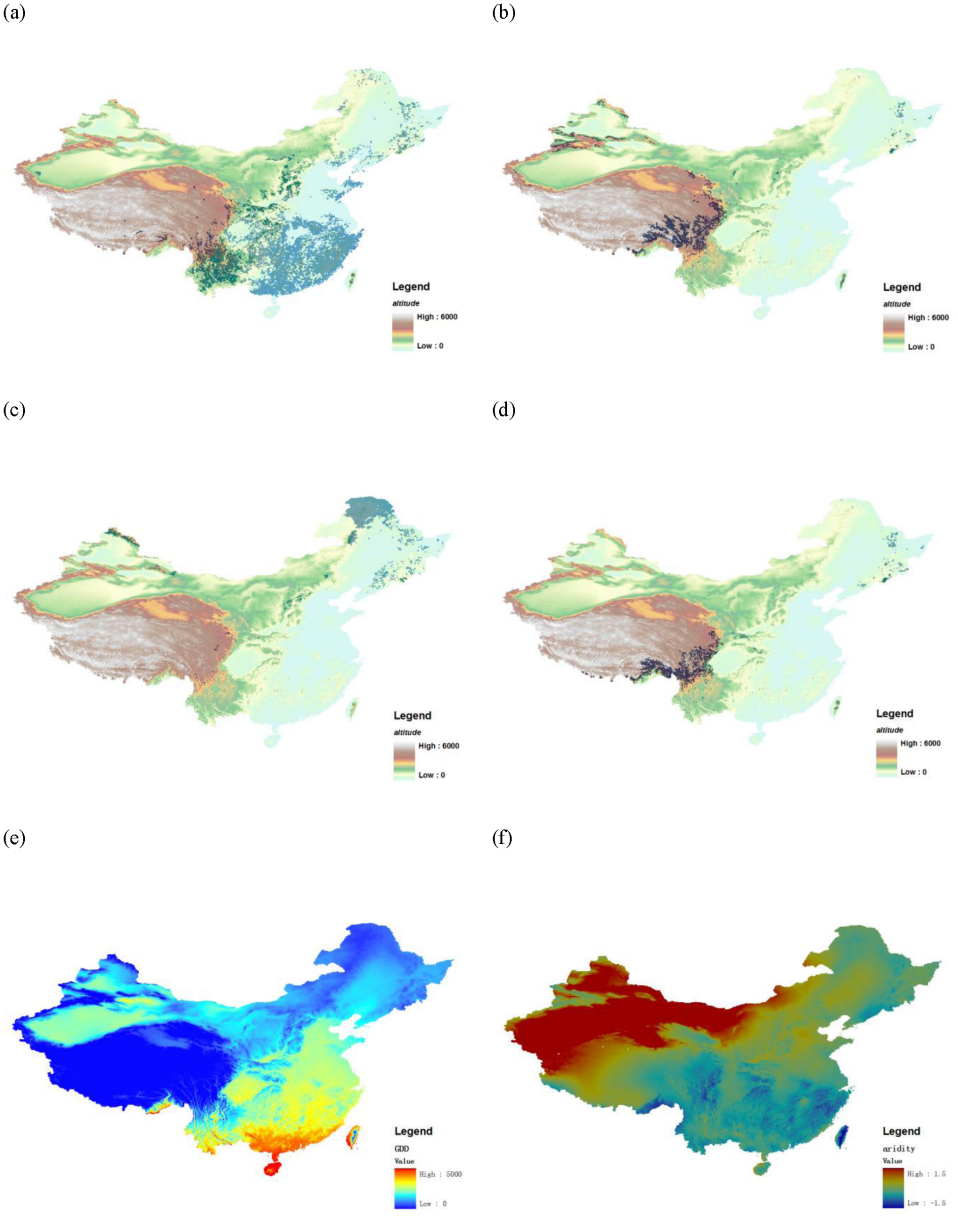
Species distributions and environmental layers. Figure (a–d) are distribution maps of species pooled to genera *Pinus*, *Abies*, *Larix* and *Picea*, respectively, superimposed over background digital elevation model (DEM) maps with 50% transparence. Figure (e) and (f) are maps of the environmental factors gross degree days (GDD) and aridity respectively.

Based on these criteria, twelve *Abies* species, twelve *Picea* species, six *Larix* species and sixteen *Pinus* species (summing up to 46 tree species) were selected for modeling as quantitatively relevant and representative species out of the total of 130 Chinese Pinaceae species. General information on the prevalence and centroid locations for all species is shown in Figure 1 and listed in Table S1.

#### Environmental data

We extracted the predictive dataset from BioPlant (download from http://www.zoology.ubc.ca/~qinli/BioKou/), a world plant bioclimatic dataset with a 10′ (latitude/longitude) sample resolution [40] and downscaled it to 5′ using change factor downscaling techniques. The BioPlant dataset calculates 15 layers of variables derived from monthly temperature and precipitation data, which were downscaled from general circulation model (GCM) predictions. Change factor downscaling techniques were adopted to obtain a fine scale dataset [41], which fully considered the effects of elevation on climatic variables.

Climatic data for calculating bioclimatic variables representing the present were downloaded from the WorldClim dataset [42], which has a resolution and interpolating schedule consistent with those used for our future scenarios. The same procedure was applied to calculate the 15 plant biological variables. Aridity was defined as the natural logarithm of the quotient of total potential evapotranspiration to total precipitation in the growing season (monthly temperature greater than 0°C in this case). Because the original BioPlant dataset did not contain this layer, we calculated it using the GRID module in ArcInfo Workstation v8.3. The time period in this study was set to the end of this century, and the future environmental layers were the 20-year means (from 2081 to 2100).

### Species Distribution Model (SDM) implementation

There are various methods of creating an ensemble of single models to reduce prediction uncertainties. In this paper, we emphasize General Circulation Model (GCM) induced and predictor selection-caused uncertainties. We used MaxEnt to build the ensembles without taking into account uncertainties related to modeling techniques.

Each ensemble was composed of 25 combinations from five GCM's and five predictor sets. The five GCM's were extracted from twenty-three GCM-derived climatic predictions from the BioPlant data set. The five GCMs were EH5, HAD, IM3, MER and PC1, of which MER and PC1 predicted the highest and lowest temperature rise, EH5 and IM3 predicted the most divergent local patterns of annual precipitation, and HAD predicted intermediate temperature and precipitation [40].

For predictor selection, we choose the most ecologically relevant bioclimatic variables based on general plant distribution theory and our understanding of the possible factors limiting Pinaceae distribution [34], [43], [44]. The five predictor sets were 1) GDD0, and Aridity; 2) T_mean, P_total, and Aridity; 3) T_cold, GDD0, and Aridity; 4) T_mean, P_total, P_season, TP_syn, and Aridity; and 5) T_cold, GDD0, P_season, TP_syn, and Aridity. In the predictor sets, GDD0, Aridity, T_mean, P_total, T_cold, P_season, TP_syn represent growing degree days above 0°C, aridity of growing season, annual temperature mean, annual total precipitation, mean temperature of the coldest month, precipitation seasonality, and synchronicity between temperature and precipitation, respectively [40].

There are considerations on the spans of predictors: the number of predictors (2, 3, and 5), whether the variables represent annual mean or seasonal extremes (Tmean vs T_cold), temperature or precipitation constraints, and whether seasonal patterns are important (P_season, TP_syn). It is evident that aridity is a major factor limiting Pinaceae distribution, thus this variable is always included in the predictor sets.

Ensembles were built for all species and scenarios (integrating 5 GCMs plus 5 predictor sets for each species). MaxEnt was used to build individual SDM's for the current distribution of each species. Then the predicted future occurrence values based on the different climate models was summarized by the median. This provided the consensus prediction of the ensemble on a cell-by-cell basis [10]. We adopted logistic output format, and the default settings for all model parameters in MaxEnt (version 3.31). Scripts were compiled to perform a batch run with MaxEnt [45], [46].

### Measurements of model performances

The model performances were measured by the area under the receiver operating characteristic curve (AUC), maximum Kappa value (*Max κ*), and the maximum true skill score (*Max TSS*) for each species. All *κ* and *TSS* values were calculated with a series of thresholds ranging from 0.05 to 0.95, and having an interval of 0.05. The *Max κ* and *Max TSS* were selected from those values for each species. Arc Macro language (AML) (in ArcInfo Workstation version 8.3) scripts were adopted to implement the calculations. Means and standard deviations of AUC, *Max κ* and *Max TSS* indices were aggregated to the genera level (*Abies, Picea, Larix*, and *Pinus*).

### Definitions for the Fuzzy Set based Potential Range Shift (F-PRS) set of indices and graphical representation

#### Threshold-free range shift indices

To quantify trends in range changes, two indices were used to measure the fundamental aspects of range shifts. The range increment index (I) measures the change in potential range area under a future climate scenario compared with the current climate. A negative value indicates range contraction and a positive value indicates range expansion. The range overlap index (O) measures the proportion of range overlap under a future climate scenario with the range overlap under the current climate. A value of 0 indicates that no overlap exists and a value of 1 indicates complete congruence. The translocation of the species range center is measured by three indices: Dx, Dy, and Dz, with the first two representing horizontal movements along longitudinal and latitudinal directions, and Dz representing the vertical movement.

The definition of a specific species range can be ambiguous when predictions are made in terms of continuous values, such as suitability or probability of occurrence [1]. Arbitrary threshold selection or selecting the ‘best threshold’ may cause incongruence in what constitutes a range, resulting in deviating predictions [23], [26], [27], [47].A threshold-free range definition based on fuzzy set theory avoids this problem. Fuzzy set theory does not perform a discrete classification among members of a data set. Actually, it numerically estimates how well a member fits into certain categories. Thus, a member can belong to several categories to some degree as measured by its memberships [48].

This study focuses on how many cells could be classified as having a specific species listed as “present.” A cell is not defined as ‘belonging’ or ‘not belonging’ to the range but rather partially belongs to the range with a degree of membership [8], [29]. Membership is a function of suitability or probability of occurrence, respectively. Here, I and O indices based on the membership function were applied [29]. The equations to calculate these indices are:
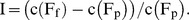


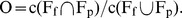
where, c represents fuzzy set cardinality, F_p_ represents the fuzzy set of the current distribution, and F_f_ is the fuzzy set of the future distribution. The indices were calculated using AML scripts in ArcInfo Workstation (Appendix File S1).

The calculated species range movements were based on the movement of the centers of each species range between time periods, where the range center was defined as the geometrical centroid of the entire potential range. To avoid the threshold selection problem, a fuzzy set style centroid was defined, instead of a traditional centroid, which did not depend on partitioning between “present” or “absent”. All cells in a grid map that contributed to the centroid were weighted by the membership of those cells that pertain to a specific species. Cells with a membership of zero do not actually contribute to the centroid but can be dealt with in the same way as any other cell.

The three dimensions (*x*, *y*, *z*) of the centroid can be calculated by:
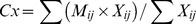


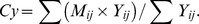


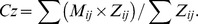
where *Cx*, *Cy*, and *Cz* are the *x*, *y*, *z* coordinates of the centroid. The *i* and *j* are the column number and row number of a cell in a grid map. *X_ij_*, *Y_ij_*, and *Z_ij_* are the coordinates of the *cell_ij_*, and *M_ij_* is a binary variable indicating whether a focal species is present or absent in *cell_ij_*. ∑ are the sums of all cells within a map.

Below, *Dx*, *Dy*, and *Dz* represent east/west, north/south, and higher/lower elevation movements, respectively. The range movement indices were defined as:
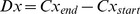


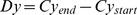


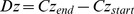
where the subscripts *end* and *start* were attached to the coordinates of centroids to indicate time periods.

In this study, membership is set equal to the logistic output of MaxEnt. Here, the units of *Dx* and *Dy* were angular degrees (longitude and latitude, respectively), and the unit of *Dz* was meters a.s.l.. All calculations were completed using ArcInfo Workstation version 8.3 (Appendix File S2).

#### Potential Range Shift chart (PRS_Chart) tool

**Figure 2:**
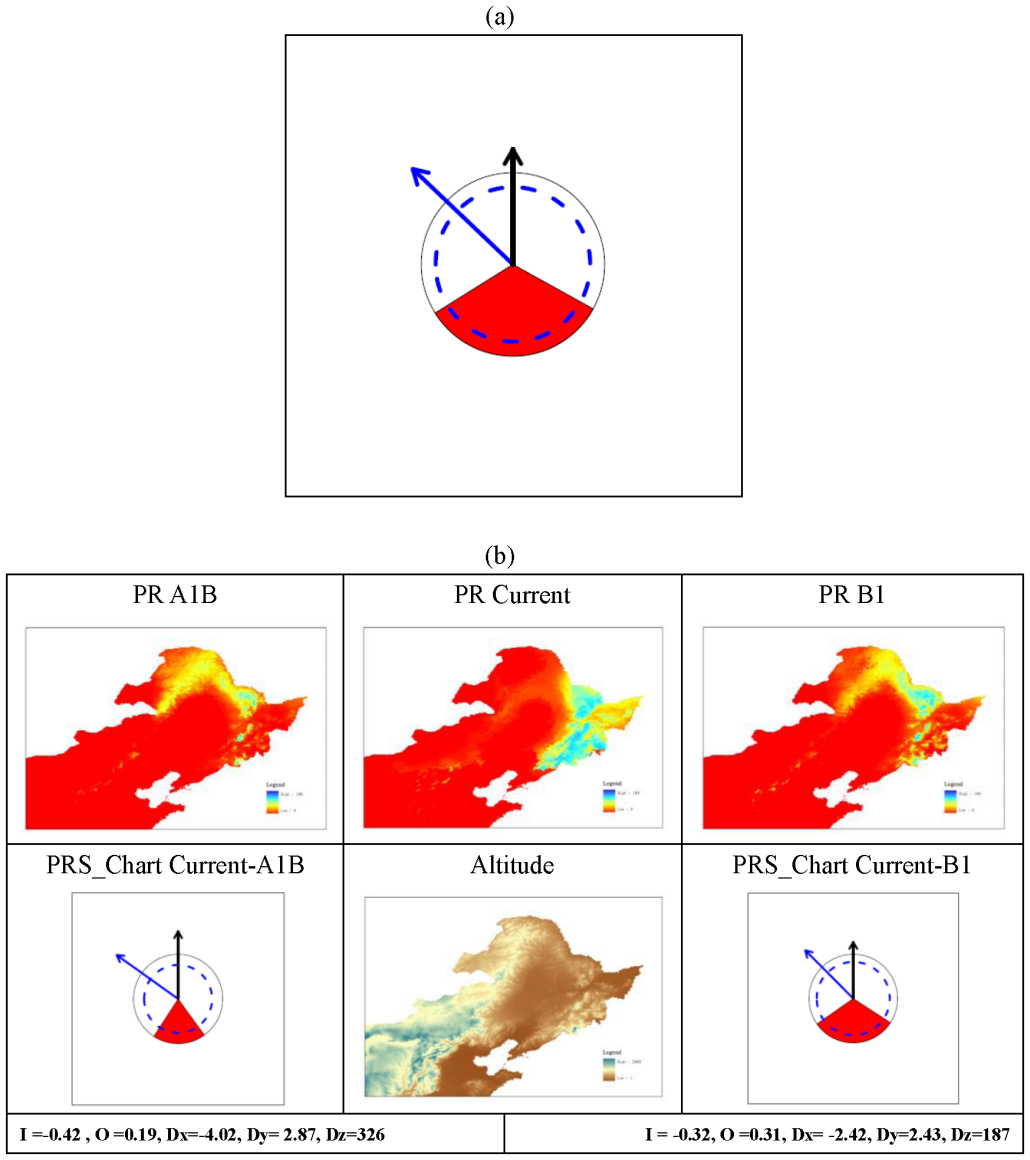
PRS_Chart and an example for *Pinus koraiensis*. Figure (a) is an example potential range shift chart (PRS_Chart) to visualize PRS indices. A unit circle (with thin black continuous line) was used to represent current distribution area for the focal species, and another circle (with broad blue broken line) with radium proportional to the ratio of distribution areas between current and projected future distributions was used to represent future distribution area. A sector of the unit circle (in red) with proportional area equal to the **O** value was used to represent range overlapping. **Dx** and **Dy** combined were represented by a directional arrow line (in blue). The distance and direction of vertical movement of range center (**Dz**) was represented by a vertical arrow (in black). Figure (b) are maps and PRS_Charts that served as an example for spices *Pinus koraiensis*. Maps in first row are potential range maps for climate scenario A1B (in the end of this century), current climate, and B1, respectively. The map in the middle of second row is digital elevation map, and charts in the left and right are PRS_Charts for A1B and A2 respectively. The values listed in bottom row are values of the PRS indices for A1B and B1.

A diagram tool named Potential Range Shift chart (PRS_Chart) (Figure 2) was developed to illustrate the five indices of Potential Range Shifts (I, O, Dx, Dy, and Dz). A unit circle was used to represent current distribution area for the focal species, and another circle with a radius proportional to the ratio of distribution areas between current and projected future distributions was used to indicate the future distribution area. Thus, I value was represented by (Area_black_ – Area_blue_)/Area_black_. A sector of the unit circle with proportional area equal to the O value was used to demonstrate range overlap.

Dx and Dy combined were represented by a directional arrow line. The direction of the arrow represents the actual movement direction of distribution centers between current and future climates. The length of the arrow tail represents the distance of the movement. In order to depict a larger range of movement distance in the chart, logarithm transformation can be applied. In this study, a base 10 logarithm transformation was used, and the unit length of the arrow represents 1.0 degree (latitude and longitude) of movement. In this manner, a 10 degree movement was drawn 2 units long. Similarly, the distance and direction of vertical movement of the range center (Dz) was represented by a vertical arrow. It is also recommended to conduct a logarithm transformation to movement distance. A base 10 logarithm transformation was used with a unit length representing 100 meters of vertical movement.

An R script file was developed to draw the PRS_Chart (Appendix 2). Range shift charts of all combinations of species and climate scenarios were drawn using this script file (Appendix File S3).

### Statistical analyses

The mean and standard deviation of I and O indices were calculated for each genus and for the three climate change scenarios under consideration. The mean and standard deviation were also calculated at the family level for each scenario. Grand means and standard deviations were finally calculated by pooling all species and scenarios.

Double-sided student distributions were used for all means of I, Dx, Dy, Dz values to test if these indices differed significantly from zero (α = 0.05). Analysis of Variance (ANOVA) testing was conducted to determine whether the difference of the five indices among scenarios was significant (α = 0.05). If any of the ANOVA analyses were significant, the least significant difference t-test (LSD-t) test was conducted for scenario to scenario comparisons (α = 0.10). This test was applied to all four genera and to the whole Pinaceae family by pooling the four genera together. All calculations were conducted in Microsoft Visual Fortran (with IMSL Stat Library).

## Results

### Performances of the SDM modeling

The high AUC values illustrate very good discriminating power of the applied models. The lowest mean AUC value among the four genera, found in the genus *Pinus*, reached 0.975. Standard deviation was generally low (with highest standard deviation of 0.018, also in *Pinus*) (Table 1). The MaxTSS values were slightly lower than those of AUC (with lowest mean MaxTSS of 0.897 in *Pinus*) and were more variable (with highest standard deviation of 0.066 in *Pinus*) (Table 1). *Max κ*, however, indicated only fair performances (with the lowest mean of 0.371 in *Abies*, and highest standard deviation of 0.180 in *Pinus*) (Table 1), although it should be noted that the scales for AUC and *MaxTSS* are from 0.5 to 1.0, representing completely random prediction to perfect prediction, while the scale for *Max κ* is from 0.0 to 1.0.

**Table 1:** Performance of SDM modeling measured by model evaluation indices.

	*Abies*	*Picea*	*Larix*	*Pinus*
**AUC**	0.984 (0.009)	0.983 (0.012)	0.982 (0.005)	0.975 (0.018)
**Max TSS**	0.935 (0.036)	0.913 (0.053)	0.920 (0.029)	0.897(0.066)
**Max ** ***κ***	0.371 (0.096)	0.375 (0.140)	0.372 (0.150)	0.380(0.180)

Notes: Table content is means and standard deviations of model evaluation indices of species distribution model (SDM) aggregated from all combinations species within the genra and climate scenarios (A1B, A2, and B1). **AUC** is area under the receiver operating characteristic curve. **Max TSS** is maximum true skill score. **Max** ***κ*** is maximum Kappa value.

### General patterns of Potential Range Shifts (PRS's)

**Figure 3:**
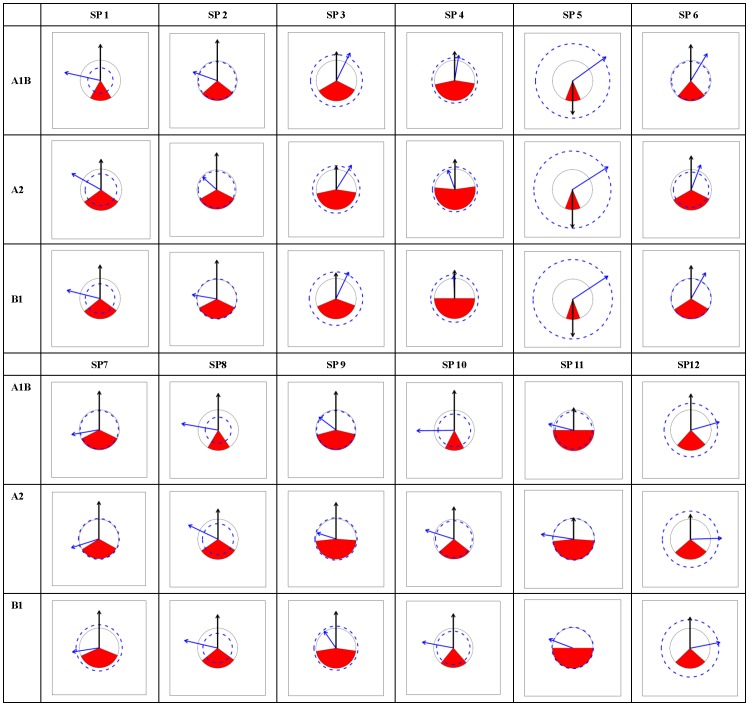
PRS_Chart for *Abies*. Each chart is a Potential range shift chart (PRS_Chart) that is explained in Figure 2. Charts in the tabular style figure representing combinations of species (SP1-12) in *Abies* and climate scenarios (A1B, A2, and B1). General species information can be found in Table S1.

**Figure 4:**
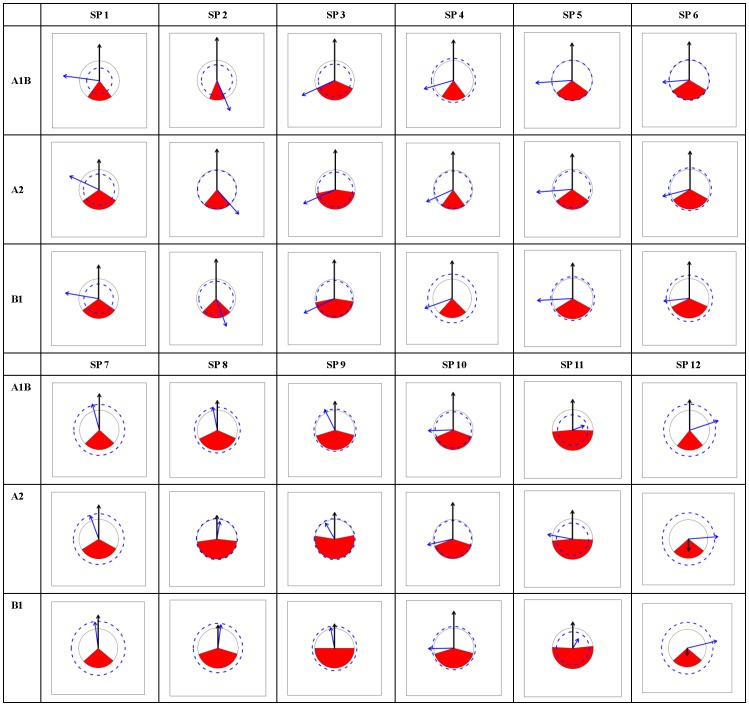
PRS_Chart for *Picea*. The same as legend of Figure 3 except the species number is for *Picea*.

**Figure 5:**
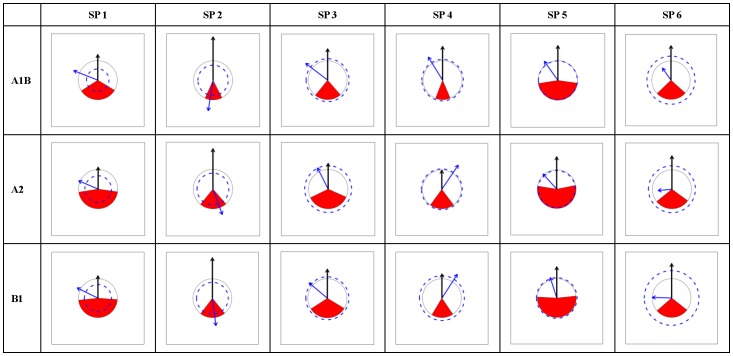
PRS_Chart for *Larix*. The same as legend of Figure 3 except the species number is for *Larix*.

**Figure 6:**
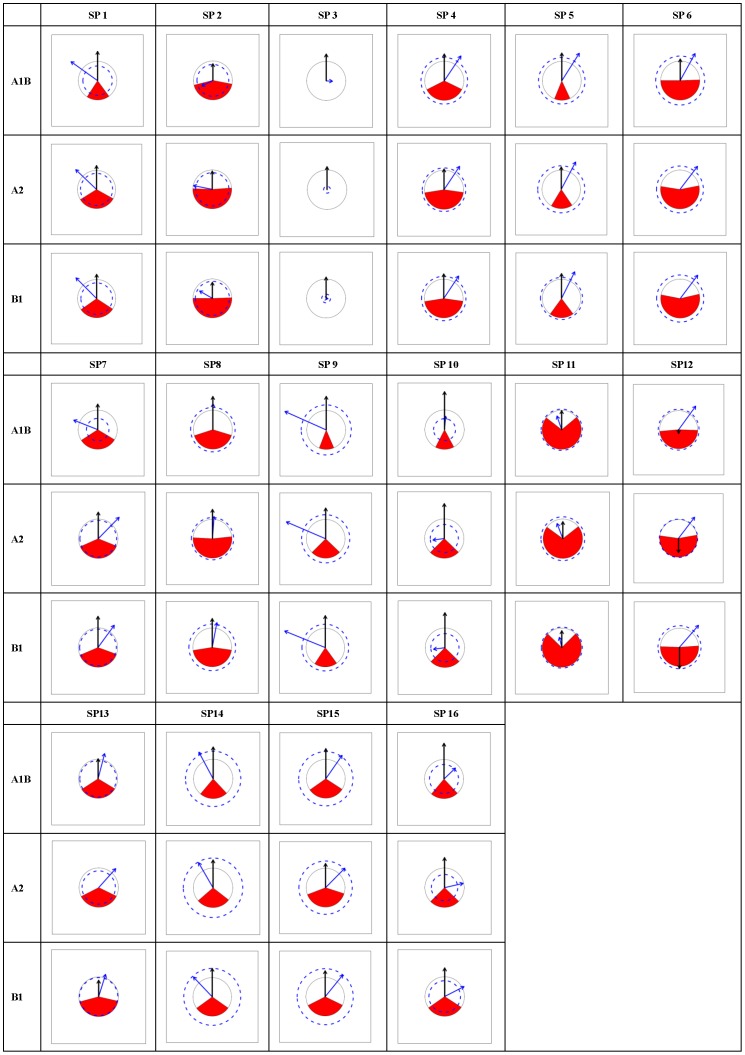
PRS_Chart for *Pinus*. The same as legend of Figure 3 except the species number is for *Pinus*.

According to the PRS_Charts (Figure 3, 4, 5, 6), the following general traits of spatial patterns can be detected:

1. Considerable interspecies variation exists in PRS's for each of the five indices;
2. PRS indices were very consistent among the three climate change scenarios for all species.
3. No clear inter-genera differences appeared in all five indices.
4. No clear range expansion or contraction trends could be detected in the I index.
5. The values of O index were generally low, mostly below 0.5 and peaking around 0.3 to 0.4. The A1B scenario was related to smaller or similar O values compared to those of A2 and B1.
6. As to horizontal movements (Dx, Dy), there was a general trend for northward spreading, but no clear trend in eastward or northward direction. Thus, the prevalent moving directions were northeast, north, and northwest.
7. As to vertical movement (Dz), almost all species moved upward with very few exceptions.
8. Species that moved south were always accompanied by exceptionally strong upward shift (large Dz), indicating a compensatory effect between upward and northward movement trends.

### Quantitative summarization of Potential Range Shifts

The mean range size changes (I index in table 2) for Pinaceae species were relatively small (0.09, 0.12, and 0.22 for A1B, A2, and B1 respectively) in comparison with large standard deviations (0.59, 0.59, and 0.63 for A1B, A2, and B1). No significant (α = 0.05) range size changes were detected both at family level and genus level for all three climate scenarios.

**Table 2:** Statistics of potential range shift indices.

Shift Indices	Emission Scenarios	*Abies*		*Picea*		*Larix*		*Pinus*		Family pooled	
		Mean	Std	Mean	Std	Mean	Std	Mean	Std	Mean	Std
**I**	**A1B**	0.17	0.82	0.02	0.42	−0.03	0.45	0.09	0.58	0.09	0.59
	**A2**	0.26	0.85	0.03	0.37	0.00	0.40	0.11	0.57	0.12	0.59
	**B1**	0.37	0.91	0.23	0.45	0.13	0.53	0.15	0.56	0.22	0.63
**O**	**A1B**	0.29	0.12	0.30	0.10	0.25	0.12	0.29	0.18	0.29	0.14
	**A2**	0.35	0.11	0.36	0.11	0.35	0.14	0.38	0.17	0.36	0.13
	**B1**	0.34	0.11	0.36	0.10	0.33	0.14	0.37	0.16	0.35	0.13
**Dx (Degree)**	**A1B**	−0.97	4.53	−1.87	3.14	−0.19	0.71	−0.72	5.28	−1.15	4.12
	**A2**	−0.17	4.14	−1.51	2.84	−0.08	1.14	−0.44	4.15	−0.60	3.52
	**B1**	−0.30	4.11	−1.41	2.80	−0.36	1.14	−0.61	4.72	−0.70	3.72
**Dy (Degree)**	**A1B**	1.56*	1.85	−0.13	1.82	0.42	2.09	2.42**	2.14	1.27**	2.18
	**A2**	1.45*	1.94	−0.25	1.52	0.52	1.64	1.87**	1.65	1.03**	1.86
	**B1**	1.51*	1.83	0.00	1.52	0.46	1.71	1.94**	1.72	1.13**	1.84
**Dz (Meters)**	**A1B**	526**	424	720**	380	669*	510	329**	238	527**	392
	**A2**	295*	408	542**	364	419*	379	188**	168	339**	345
	**B1**	357*	412	528**	351	472*	398	205**	173	364**	342

Notes: Table contents are mean(**mean**) and standard deviation (**Std**) of potential range shift (PRS) indices representing by combinations of **I**, **O**, **Dx**, **Dy**, **Dz** and CO_2_ emission scenarios **A1B**, **A2**, and **B1** shown in first two columns. Superscripts ** after mean values indicating that the mean is very significant from zero (α = 0.01), and * is significant different from zero (α = 0.05) by using double sided student distribution. No superscript after mean value means it is not significant from zero, except the mean of O index which did not test for deviation from zero.

The range overlapping (O index in Table 2) showed low values (with means 0.29, 0.36 and 0.35 for A1B, A2, and B1) and relatively small interspecific variations (with standard deviations 0.14, 0.13 and 0.13 for A1B, A2, and B1) at family level and a similar pattern at the genus level.

The longitudinal location of the range center (Dx index in Table 2) was found to move slightly westward (with mean −1.15, −0.60 and −0.70 degrees for A1B, A2, and B1) but this trend was not statistically significant and was connected with large interspecific variation (standard deviation 4.12, 3.52, and 3.72 degrees for A1B, A2, and B1). Comparable results were found for all genera.

The latitudinal displacement of the range center at the family level (Dy in Table 2) was slightly larger (with mean1.27, 1.03, and 1.13 degrees for A1B, A2, and B1) than the longitudinal trend, but had much smaller interspecific variations (with standard deviation 2.18, 1.86, and 1.84 degrees for A1B, A2, and B1). The northward movement was highly significant (α = 0.01) at the family level. Comparable trends were found in *Pinus* and *Abies* but did not emerge in *Picea* and *Larix*. This indicates that the northward trend at the family level can be attributed mainly to pine and fir species.

The vertical movement (Dz in Table 2) trends were highly significant at the family level and were also highly significant in *Pinus* and *Picea*, but the significance of *Abies* and *Larix* depends on climate scenarios. Mean movement distances of the family were 727, 339, and 364 meters, with standard deviations 392, 345, and 342 meters for A1B, A2, and B1, respectively.

### Inter-scenario comparisons

The ANOVA (Table 3) indicated no significant difference (α = 0.05) among scenarios in horizontal directions (Dx and Dy) both at the genus and at the family level. There was also no significance among scenario differences in range size changes (O index) at family and genus level with the exception of *Picea*, which responded to scenario B1 with a significantly (α = 0.10 by LSD-t test) larger range expansion compared to A1B or A2.

**Table 3:** Comparisons of Potential range shift (PRS) indices among climate scenarios.

Shift Indices	Tested Item	*Abies*	*Picea*	*Larix*	*Pinus*	Family pooled
**I**	**ANOVA**	X	P = 0.032	X	X	X
	**A1B**	/	0.02^a^	/	/	/
	**A2**	/	0.03^a^	/	/	/
	**B1**	/	0.23^b^	/	/	/
**O**	**ANOVA**	P = 0.034	P = 0.007	P = 0.018	P = 0.010	P = 0.000
	**A1B**	0.29^a^	0.30^a^	0.25^a^	0.29^a^	0.29^a^
	**A2**	0.35^b^	0.36^b^	0.35^b^	0.38^b^	0.36^b^
	**B1**	0.34^b^	0.36^b^	0.33^b^	0.37^b^	0.35^b^
**Dx**	**ANOVA**	X	X	X	X	X
	**A1B**	/	/	/	/	/
	**A2**	/	/	/	/	/
	**B1**	/	/	/	/	/
**Dy**	**ANOVA**	X	X	X	X	X
	**A1B**	/	/	/	/	/
	**A2**	/	/	/	/	/
	**B1**	/	/	/	/	/
**Dz**	**ANOVA**	P = 0.030	P = 0.026	X	P = 0.000	P = 0.000
	**A1B**	526^a^	720^a^	/	329^a^	527^a^
	**A2**	295^b^	542^b^	/	188^b^	339^b^
	**B1**	357^b^	528^b^	/	205^b^	364^b^

otes: The table contents after row **ANOVA** are results of analysis of variance for each genus comparing among climate scenarios, in which X representing not significant (α = 0.05) or P-values is provided significant. Contents after row **A1B**, **A2**, and **B1** are results of multiple comparisons if the ANOVA result is significant by using least significant difference t test (LSD-t). the/means test is not carried out. Number and superscript represent mean value and multiple comparison result, with same superscript characters meaning not significant and different character meaning significant (α = 0.10).

Generally, there were highly significant differences among scenarios (α = 0.10 by ANOVA in Table 3) in range overlap (O index) and vertical movement (Dz) at family level. O values in A1B scenario were found to be significantly smaller than those in A2 and B1, and Dz values in A1B were significantly larger than those in A2 and B1. A similar pattern was also found at the genus level but with a few cases of ANOVA results being significant instead of highly significant. An exception was the genus *Larix*, which did not differ in vertical movement among scenarios. Consistent lower O values and larger Dz values indicated that a realization of scenario A1B would lead to the strongest range shifts.

## Discussion

### Performance of the novel set of indices for tree species range shift

Modeling results on the future potential optimum distribution of tree species are urgently needed for forest ecosystem management and silviculture in order to avoid the loss of resources and ecosystem services. Climate change projections are still vague to a certain degree mainly due to uncertainties in global economic development, mitigation policy, and technological progress. However, general trends emerge especially when scenarios are compared.

Here, a new quantitative index is presented as a tool for forestry, ecology, and biogeography that circumvents restrictions and disadvantages of former species distribution modeling approaches according to their focus on selected species, scenarios and directions, and intensity of shift. This research contributes to the rapid development and progress of biogeographical modeling in response to the ongoing global changes [49], [50], [51], [52], [53], [54]. In this study, models are based on data for grid cells where the species were common or even dominant in order to avoid bias from small species populations on special sites with extraordinary microclimatic conditions that were not representative for the climate at the landscape scale of the modeled grid cells. Distributional data of this type did not differ substantially from total species ranges inferred from other map sources [39], [55].

As a novel and robust approach, the Fuzzy Set based Potential Range Shift Index (F-PRS Index) is introduced in this study. Here, the application is focused on dominant, widespread, and economically important conifer species of the Pinaceae family in China. However, the method can be implemented to other species groups and regions in the future.

The F-PRS Index: (1) helps detect patterns that hide behind large amounts of information, and thus deepens understanding of the studied phenomena; (2) delivers a powerful tool for the building of statistically testable hypotheses; and (3) supports the efficient implementation of meta-analyses. Here, the novel F-PRS Set of Indices performance is demonstrated according to the first two aspects.

One important piece of information on the upward movement trends becomes evident by the Dz index, which integrates three levels of spatial information (current distribution, future distribution, and altitude). It is expected that this will contribute to the third F-PRS Index aspect, and delivers an excellent basis for meta-analyses after wider applications.

General requirements for indices are: (1) The ability to extract key attributes that are essential to the studied phenomena out of complex interactions and processes; (2) The capacity to reach standardization and comparability among studies; (3) The stability against random perturbations; and (4) The independence of the members of the indices set.

The F-PRS indices set introduced in this study definitively fulfills the first criterion because the five aspects represented by the five member indices are all critical attributes for range studies. Species range size change is one of most concerning topics in climate change biology [56], [57]. Range size conservation, and range size being identified as species level trait to test species level selection have instigated a large number studies in evolutionary ecology [58], [59]. Range overlapping that measures the ratio of area shared by the current range and future range has a profound application in conservation biology [60]. Range position change is a hot topic in biogeography.

The drivers of northward and upward movements for species in response to climate warming are intensely studied both empirically and theoretically [61], [62], [63], [64], [65], [66]. The temperature related “altitude for latitude” movement hypothesis is intensely debated in biogeography [67]. However, longitudinal movement, for instance in response to changes in precipitation regimes, is a little touched topic due to a lack of methodological concepts. Here, a tool that enables the detection of anisotropy in temporal trends in all directions is presented.

One preeminent advantage of the F-PRS indices set is that they are standardized and can be adopted to make comparisons among different studies. Although it requires that the SDM's predicted suitability maps are consistent, which is not granted in all cases, it can at least be used to make comparisons among studies using the same SDM. Ensemble models that integrate various species distribution modeling algorithms such as BIOMOD can serve as a standard pre-analysis in order to avoid deviations between specific SDM's [13].

The fuzzy set style indices presented here are more stable than former threshold defined measurements of range shifts [29]. Two sources of mechanisms may produce unstable results for threshold measurements. The first one is that small perturbations (such as random processes during SDM modeling and different strategies in selecting pseudo absent data) may cause large differences in selecting “best” threshold values, thus resulting in deviating range maps. The second source of uncertainty in traditional approaches comes from the fact that even small perturbations in threshold can cause large differences in range maps in case that the predicted suitability map contains a large area of very low values. The fuzzy set indices are certainly free from the first source of instability, because they are threshold free, and performed much better in dealing with the second source [29].

Lastly, it is evident that all the five indices in the F-PRS indices set have clearly differentiated meanings. Thus, we believe the new F-PRS indices set is good based on the above criterions.

### Applicability in range change studies and forest managements

In practice, it is critical to achieve mutual understanding between researchers and policy makers via readily available communication techniques. Visualization is one of the most emphasized strategies to facilitate communication, the exchange of information, and its implementation [30]. In this case study, a graphical representation of range shifts (Figure 3, 4, 5, 6) is introduced, which will contribute to an efficient climate adapted management policy for coniferous forest in China. The effects that climate change is likely to bring about to Chinese forests can be instantly recognized. Consequences for climate adapted management becomes obvious.

Visualization is not only beneficial for forest management but also for research. Although it seems that all the information presented in the graphs has already been contained in the calculated indices, all the spatial information that is emerging in the patterns may not be revealed from numerical indices. Graphs are more appropriate than single values or numbers to demonstrate large-scale spatial patterns, which is exemplified by the eight general patterns identified in this study (section3.2). Based on these spatial findings, more specific hypotheses can be raised to be tested in the future.

Unlike theoretical studies, which usually emphasize summarizing general patterns and further reveal underlying mechanisms that form a large amount of case studies [68], [69], [70], [71], management sectors such as forestry and conservation biology need to keep balance between generality and detail [72], [73]. At one end, overall policy to maintain forest ecosystems is highly emphasized, at the other end, not a single species can be neglected in forestry or nature conservation. As the former has been fully emphasized, it is necessary to draw some attention on details, especially a few exceptional cases that can be clearly seen from the graphs (Figure 3, 4, 5, 6). For example, AbiesT05 (*Abies spectabilis*) had exceptionally large range expansion (several times the current range) and showed a strong downward movement trend in each scenario. PinusT03 (*Pinus pumila*) seemed to lose almost its complete potential range in each of the scenarios. PinusT11 (*Pinus massoniana*) had very large overlap between scenarios and little movement, indicating a stable range position. *Picea* speciesT11 (*Picea morrisonicola*) had very similar horizontal movement trends between scenarios A1B and B1, but was very different in A2, and PiceaT12 (*Picea spinulosa*) displayed large upward movement in A1B but moved slightly downward in A2 and B1. Apart from the fact that knowing the reasons that brought about those exceptions might have theoretic significance, knowing the facts themselves also has conservation and management importance.

### Assisted migration of tree species in climate adaptive forest management

The results of this study show that for various species with considerable range displacement, assisted migration becomes more or less inevitable in order to maintain the functioning of ecosystems and to preserve the biological diversity of tree species. Intended translocation of endangered species has been proposed to avoid climate change caused extinction [74]. Immediate concerns about unintended side effects were expressed [75], [76] and debates were aroused [77], [78], [79], [80], [81].

Here, extending the idea of assisted migration and endangered species translocation in biological conservation is proposed for the rearrangement of a larger amount of, as well rare, economically important tree species. However, the design of “novel ecosystems” is currently under debate and ecological and practical consequences are not solved, yet. Based on this case study on Pinaceae species in China, there are only around one third of overlapping areas that are both suitable for the species at current climate and under climatic conditions in the end of this century, not accounting for species adaptive ability and other sources of uncertainty.

The huge overall changes in suitable ranges require that foresters track the potential ranges of most of the Pinaceae species, otherwise a tremendous decline of coniferous forest cannot be excluded. It is unfeasible and unrealistic to let natural processes (i.e. nature dispersal or local adaptation) fulfill this task for two reasons: firstly, current speed of anthropogenic caused climate change is much faster than natural climate variability in history [82], thus this will leave no chance for tree species to adapt in a short time period [83]. Secondly, the high fragmentation of natural habitats segregated by agricultural land uses, urban areas and road systems is suppressing dispersal and colonization of new habitats [84], [85], [86]. More broadly, forest management needs to identify and prepare in advance habitats for the establishment of larger numbers of the tree species close to but perhaps outside their previous “native” range, or even novel forest communities because they were formerly considered to be potentially natural communities, not representing future climate anymore [87].

Fundamental changes in forestry will become unavoidable. Although there are many risks connected to the introduction of species to new localities outside of their previous distribution, such as biological invasion or unwanted species interactions, keeping all species in their current locations will not be unfeasible either. In this study it isshown that about two-thirds of the coniferous forests in China may experience species loss and become restricted in their functioning.

We support the point made by Thomas [81] that the question is not on whether or not to make changes, but on how to manage forests in a way that promotes advantages and avoids disadvantages. This involves a broad and deep understanding of the processes and the functioning of ecosystems. Thus, the idea of climate adaptive management requires not only best practice and reliable projections for species range shifts, but also the integration of knowledge of all basic branches of biological science, such as ecology, biogeography, genetics and evolution.

### Limits and perspectives

Species distribution models and climate envelope modeling features some intrinsic limitations, such the disregard of evolutionary processes and adaptation, or the ignorance of within-species genotypic and ecological variability. Experimental research must be designed to specifically address these issues that cannot be tackled by the actual nature of species distribution models. However, in the case of key tree species, rapid directives are needed in order to adapt species composition and structure of forests at an early stage to the environmental conditions that are expected in the near future during the life-time of the planted saplings. Evolutionary processes are irrelevant at this time scale. However, it is important to know genetic and ecological variability, especially for tree species with a large spatial distribution [88].

The introduced set of indices delivers a powerful and practical approach for adapting the Chinese forests to future developments that are forecasted in climate scenarios and global climate models. Uncertainty according to realized scenarios and climate model output must be considered in forest management [89]. Especially the role of climatic extremes that are expected to become more and more frequent and intense (IPCC 2012) is an issue that is important in forest practice but not satisfactory in its reflection in climate models. Climatic extremes are much more important for long-lived species such as trees than long-term trends in average values [90].

In consequence, flexible approaches are asked for which allow management to be adapted for the developing state of knowledge, to further develop societal and emission scenarios, and to improved climatic projections. The introduced set of indices, which can be applied for larger groups of tree taxa, offers these options and will allow the implementation of new findings, in order to identify their relevance in forestry.

In perspective, it is not only the application of such models but also the assignment and upscaling of species distribution models to global vegetation models is required. Key species for important biomes, such as the members of the Pinaceae family, are neglected in the current state-of-the-art approaches for global vegetation models that are the basis for the calculation of future carbon fluxes and budgets. The introduced approach in this study brings these modelling philosophies one step closer and may contribute to make vegetation models that integrate plant functional types such as needle-leaved trees more reliable.

## Conclusions

As a consequence of the long-term life cycles of trees, there is an urgent need in forestry for reliable forecasts on future ranges of tree species. Species Distribution Models are providing such projections but at continental scales, many species must be taken into account. The Pinaceae family represents a high number of ecologically and economically important tree species in China. For this reason, this family was chosen to develop and implement a new set of indices that enable the detection of directions and intensity of range shifts at the species, genus and family level.

This new threshold free set of indices for tree range shifts features several advantages compared to traditional approaches. Besides its capacity to integrate across a large number of species and to identify general patterns of range shifts for large species groups, the PRS_Chart graphical tool offers an intuitive visualization tool for the science-policy interface which will support the translation of research into practice.

Particularly, the predicted low range overlap between current and future tree species ranges combined with a significant northward as well as upward movement trend for the whole group of species implies that assisted migration can become increasingly important in climate adaptive forest management.

## Supporting Information

File S1 AML file for calculating PRS indices (I, O).An AML script file under ARCGIS 8.3 Workstation for calculating PRS indices I and O.
(AML)

File S2 AML file for calculating PRS indices (Dx, Dy, Dz).An AML script file under ARCGIS 8.3 Workstation for calculating PRS indices Dx, Dy, and Dz.
(AML)

File S3 R file for plotting PRS_Chart. A R script file under R version 2.15.1 for plotting PRS_Chart.
(R) Click here for additional data file.

Table S1 General information of species studied.Notes: SP.No.: species number; Prev.: species prevalence indicated by ratios*10^–4^ of cells occupied by that species to total study area; LG and LT: longitude and latitude of the species centroid.
(DOCX)
