# Supplementary material for: A New Tool for Exploring Climate Change Induced Range Shifts of Conifer Species in China
Source: PLoS One. 2014 Sep 30;9(9):e98643. doi: 10.1371/journal.pone.0098643 (PMC4181978; doi:10.1371/journal.pone.0098643)
Supplement: Table S1 — General information of species studied.Notes: SP.No.: species number; Prev.: species prevalence indicated by ratios*10–4 of cells occupied by that species to total study area; LG and LT: longitude and latitude of the species centroid. (DOCX) [file pone.0098643.s004.docx]

**Table S1** General information on Pinaceae species in China included in the study

| **SP. No** | **Scientific Name** | **Prev.** | **LG.** | **LT.** | **SP. No** | **Scientific Name** | **Prev.** | **LG.** | **LT.** |
| --- | --- | --- | --- | --- | --- | --- | --- | --- | --- |
|  |  |  |  |  |  |  |  |  |  |
| **AbiesT01** | *Abies nephrolepis* | 32.9 | 128.96 | 45.68 | **LarixT01** | *Larix gmelinii* | *260.8* | 123.85 | 50.79 |
| **AbiesT02** | *Abies fargesii* | 7.7 | 106.07 | 33.05 | **LarixT02** | *Larix sibirica* | *31.6* | 90.62 | 46.37 |
| **AbiesT03** | *Abies georgei* | 76.1 | 97.24 | 29.04 | **LarixT03** | *Larix olgensis* | *39.9* | 126.29 | 42.61 |
| **AbiesT04** | *Abies squamata* | 42.6 | 100.33 | 30.40 | **LarixT04** | *Larix gmelinii var. principis-rupprechtii* | *8.9* | 114.55 | 39.75 |
| **AbiesT05** | *Abies spectabilis* | 3.1 | 90.62 | 28.73 | **LarixT05** | *Larix potaninii var. australis* | *4.0* | 100.49 | 29.88 |
| **AbiesT06** | *Abies delavayi* | 21.5 | 99.04 | 27.49 | **LarixT06** | *Larix potaninii* | *2.2* | 104.59 | 34.04 |
| **AbiesT07** | *Abies fargesii var. faxoniana* | 32.0 | 102.95 | 32.20 |  |  | *0.0* |  |  |
| **AbiesT08** | *Abies fabri* | 21.4 | 102.55 | 29.93 |  |  | *0.0* |  |  |
| **AbiesT09** | *Abies forrestii* | 17.0 | 101.85 | 28.75 |  |  | *0.0* |  |  |
| **AbiesT10** | *Abies holophylla* | 7.8 | 126.96 | 42.13 | **PinusT01** | *Pinus koraiensis* | *65.5* | 128.07 | 44.85 |
| **AbiesT11** | *Abies kawakamii* | 2.7 | 119.95 | 24.43 | **PinusT02** | *Pinus sylvestris var. mongolica* | *21.1* | 123.29 | 49.59 |
| **AbiesT12** | *Abies delavayi var. motuoensis* | 21.7 | 94.99 | 28.87 | **PinusT03** | *Pinus pumila* | *3.4* | 122.62 | 51.87 |
|  |  |  |  |  | **PinusT04** | *Pinus tabuliformis* | *129.2* | 113.87 | 37.84 |
| **PiceaT01** | *Picea koraiensis* | 29.9 | 128.98 | 45.53 | **PinusT05** | *Pinus densiflora* | *28.6* | 120.74 | 37.17 |
| **PiceaT02** | *Picea obovata* | 7.7 | 88.76 | 47.88 | **PinusT06** | *Pinus bungeana* | *3.4* | 109.45 | 31.71 |
| **PiceaT03** | *Picea schrenkiana* | 57.2 | 83.72 | 43.19 | **PinusT07** | *Pinus armandii* | *57.1* | 106.93 | 32.52 |
| **PiceaT04** | *Picea wilsonii* | 10.5 | 103.98 | 34.16 | **PinusT08** | *Pinus densata* | *42.6* | 99.56 | 28.57 |
| **PiceaT05** | *Picea crassifolia* | 19.9 | 101.93 | 36.91 | **PinusT09** | *Pinus taiwanensis* | *23.9* | 110.73 | 29.54 |
| **PiceaT06** | *Picea asperata* | 17.9 | 103.18 | 33.28 | **PinusT10** | *Pinus tabuliformis var. henryi* | *3.6* | 108.29 | 31.84 |
| **PiceaT07** | *Picea likiangensis* | 26.0 | 100.86 | 28.64 | **PinusT11** | *Pinus massoniana* | *944.4* | 113.08 | 27.58 |
| **PiceaT08** | *Picea likiangensis var. linzhiensis* | 31.0 | 94.71 | 29.75 | **PinusT12** | *Pinus yunnanensis* | *191.9* | 102.44 | 26.07 |
| **PiceaT09** | *Picea likiangensis var. rubescens* | 134.2 | 98.87 | 30.69 | **PinusT13** | *Pinus kesiya var.langbianensis* | *34.1* | 101.17 | 23.62 |
| **PiceaT10** | *Picea purpurea* | 40.4 | 103.01 | 32.59 | **PinusT14** | *Pinus thunbergii* | *24.6* | 120.56 | 32.89 |
| **PiceaT11** | *Picea morrisonicola* | 4.6 | 121.09 | 23.67 | **PinusT15** | *Pinus palustris* | *2.7* | 91.71 | 28.21 |
| **PiceaT12** | *Picea spinulosa* | 8.6 | 92.56 | 27.67 | **Pinust16** | *Pinus yunnanensis var. pygmaea* | *6.6* | 102.80 | 28.00 |

Notes: SP.No.: species number; Prev.: species prevalence indicated by ratios*10^-4^ of cells occupied by that species to total study area ; LG and LT: longitude and latitude of the species centroid.
